# Gradient boosting and bayesian network machine learning models predict aflatoxin and fumonisin contamination of maize in Illinois – First USA case study

**DOI:** 10.3389/fmicb.2022.1039947

**Published:** 2022-11-10

**Authors:** Lina Castano-Duque, Martha Vaughan, James Lindsay, Kristin Barnett, Kanniah Rajasekaran

**Affiliations:** ^1^USDA, Agriculture Research Service, Southern Regional Research Center, New Orleans, LA, United States; ^2^USDA, Agricultural Research Service, National Center for Agricultural Utilization Research, Mycotoxin Prevention and Applied Microbiology Research Unit University, Peoria, IL, United States; ^3^Office of National Programs, Agriculture Research Service, USDA, Beltsville, MD, United States; ^4^Illinois Department of Agriculture, Agricultural Products Inspection, Springfield, IL, United States

**Keywords:** aflatoxin, fumonisin, machine learning, gradient boosting, bayesian network, Illinois

## Abstract

Mycotoxin contamination of corn results in significant agroeconomic losses and poses serious health issues worldwide. This paper presents the first report utilizing machine learning and historical aflatoxin and fumonisin contamination levels in-order-to develop models that can confidently predict mycotoxin contamination of corn in Illinois, a major corn producing state in the USA. Historical monthly meteorological data from a 14-year period combined with corresponding aflatoxin and fumonisin contamination data from the State of Illinois were used to engineer input features that link weather, fungal growth, and aflatoxin production in combination with gradient boosting (GBM) and bayesian network (BN) modeling. The GBM and BN models developed can predict mycotoxin contamination with overall 94% accuracy. Analyses for aflatoxin and fumonisin with GBM showed that meteorological and satellite-acquired vegetative index data during March significantly influenced grain contamination at the end of the corn growing season. Prediction of high aflatoxin contamination levels was linked to high aflatoxin risk index in March/June/July, high vegetative index in March and low vegetative index in July. Correspondingly, high levels of fumonisin contamination were linked to high precipitation levels in February/March/September and high vegetative index in March. During corn flowering time in June, higher temperatures range increased prediction of high levels of fumonisin contamination, while high aflatoxin contamination levels were linked to high aflatoxin risk index. Meteorological events prior to corn planting in the field have high influence on predicting aflatoxin and fumonisin contamination levels at the end of the year. These early-year events detected by the models can directly assist farmers and stakeholders to make informed decisions to prevent mycotoxin contamination of Illinois grown corn.

## Introduction

Multiple sectors of the USA agricultural industry (growers, processors, and consumers) are negatively affected by mycotoxin contamination of grain crops, with annual losses estimated in the range of $418 million to $1.66 billion (Vardon et al., 2003; Wu, 2006; Mitchell et al., 2016). Mycotoxin contamination is also a critical food safety concern as these toxins can cause severe health issues to humans and livestock (Vardon et al., 2003). Corn is susceptible to contamination by diverse mycotoxin classes; however, two of the major classes, aflatoxins (AFL), and fumonisins (FUM), are most problematic. AFL and FUM are produced by distinct fungal species of *Aspergillus* and *Fusarium via* well characterized metabolic pathways (Proctor et al., 2018; Munkvold et al., 2019). AFL contamination of corn is primarily caused by *A. flavus* and *A. parasiticus,* while the causal agents of FUM contamination are predominantly *F. verticillioides* and *F. proliforatum* (Woloshuk and Shim, 2013). Environmental conditions have a strong influence on corn mycotoxin contamination, and the optimal conditions that favor disease development and mycotoxin production vary. *Aspergillus* and *Fusarium* species can grow and produce mycotoxins at a wide range of temperatures, but the optimal growth temperatures for *A. favus* are 30–35°C (Abdel-Hadi et al., 2012), while *F. verticillioides* favors slightly lower temperatures ranging between 20 and 25 °C (Medina et al., 2014). Dry, hot conditions favor *A. flavus* conidiation and dispersal while compromising corn growth and defenses. Thus, high temperatures and drought stress are typically associated with AFL contamination (McMillian et al., 1985; Payne et al., 1986; Diener et al., 1987; Payne et al., 1988; Widstrom et al., 1990; Payne and Widstrom, 1992; Guo et al., 1996; Sétamou et al., 1997; Scheidegger and Payne, 2003; Cotty and Jaime-Garcia, 2007). *Fusarium* infection of corn and FUM contamination are also associated with warm temperatures and drought stress but kernel water activity and insect injury are additional contributing factors (Warfield Colleen and Gilchrist, 1999; Miller, 2001; Munkvold, 2003; Bush et al., 2004).

Efforts to minimize economic losses associated with mycotoxin contamination of grain include timely implementation of management and mitigation strategies in the field, during grain handling, and in storage. However, with ears concealed in the husk, corn farmers frequently do not know whether they have an ear rot disease or mycotoxin contamination problem until harvest, when it is too late for the implementation of mitigation strategies. In this regard, tools such as prediction of mycotoxin contamination and risk assessment systems are needed to alert growers and other stakeholders of possible risks of mycotoxin outbreaks. These predictive tools will provide stakeholders with a proactive window of opportunity to deploy strategies specifically aimed at controlling disease and thus grain contamination (Focker et al., 2020).

Modeling tools have demonstrated benefits in predicting mycotoxin risk internationally but do not benefit USA corn growers. In Eastern Europe, models have been developed and applied to predict the risk of mycotoxin contamination in milk (Van der Fels-Klerx et al., 2019). Additionally, models assessing mycotoxin risk in small grains, corn, and other cereal crops, have been developed in Italy (Battilani et al., 2013; Leggieri et al., 2021a), Serbia (Liu et al., 2021), Europe (Wang et al., 2022), and Korea (Lee et al., 2018). However, these models are generally not applicable, are not fine-tuned, or commercially available for corn growers in the USA because differences in weather, linked to geographical location, and historical mycotoxin contamination result in major discrepancies in model predictions and accuracy levels (de Schrijver et al., 2021). One exception is the web-based *Fusarium* head blight (FHB) risk assessment tool from the USA Wheat and Barley Scab Initiative.^1^ The USA wheat-scab initiative has implemented modeling approaches that accurately alert wheat and barley growers of FHB risk (Shah et al., 2018). However, unlike FHB of wheat or barley and associated deoxynivalenol mycotoxin contamination, AFL and FUM contamination risk of corn is not always coupled with disease symptoms. USA-based predictive models based on insurance claims as a proxy for mycotoxin contamination values are available for corn (Yu et al., 2020, 2022), and these models have provided valuable information showing that mycotoxin related crop losses will likely become more severe as temperatures increase (Yu et al., 2022). However, insurance claims are not necessarily equivalent to actual determined mycotoxin values. Corn growers will benefit from models that are trained with observed historical contamination values and risk assessments that incorporate different levels of contamination. Therefore, models developed using mycotoxin data from USA corn growing states are critically needed.

The main objective of this research was to evaluate the connection between historical weather parameters with AFL and FUM contamination by using machine learning models that could be directly utilized by industry or corn growers and would eventually serve as the basis for prediction of mycotoxin contamination risk at state-specific and potentially nationwide levels in the USA. We chose the state of Illinois as a first case study because of the availability of extensive historical data-sets. Models described herein were developed highlighting three elements from previous studies: (1) each of the non-USA based models (mainly European) were optimized for a determined geographical area considering climate variables (Battilani et al., 2016; Liu et al., 2021; Nji et al., 2022), for example, temperature, precipitation, and other factors specific to the location. (2) The modeling tools for *Aspergillus* mainly had a mechanistic basis that included biological relationships of the fungus with the environment and the plant host. (3) These relationships can be summarized as mathematical functions capable of determining fungal growth, toxin index, and fungal dispersal among other variables (Battilani et al., 2013). The advantage to us was that these functions served as the basis to create new input features that can be called engineered features (Battilani et al., 2016; Van der Fels-Klerx et al., 2019; Leggieri et al., 2021a,b; Wang et al., 2022). As such, the European-based models served as a general roadmap to model prediction of AFL and FUM contamination in Illinois.

Herein, we used 14 years of historical contamination data, combined with monthly county-wise weather data to perform analyses. Feature engineering (Battilani et al., 2013; Van der Fels-Klerx et al., 2019) was used in combination with gradient boosting models (GBM; Friedman, 2001) and bayesian networks (BN; Cooper, 1990) to predict AFL. For prediction of FUM contamination, GBM and BN were used in combination with weather and plant-related parameters.

## Materials and methods

### Mycotoxin and weather data

Historical mycotoxin contamination data for corn production was obtained from the Illinois Department of Agriculture for every county in the State for 2003–2004, and from 2007 through 2019 (data for 2020 was not available due to COVID-19 pandemic). Data from 2021 was obtained from the same source and was reserved only for model validation. Historical AFL and FUM mycotoxin survey data is available at https://www2.illinois.gov/sites/agr/Plants/Mycotoxin/Pages/Survey.aspx. The sample collection was done at random by sampling from four corn producers from each county (2.3–4.5 kg of whole kernels). Half of the collected sample were split, and 10 g were used for mycotoxin quantification. Mycotoxin quantification was performed using Watex kits from Romer starting in 2017, prior to that date quantification was done using Neogene Elisa test kits. Mycotoxin data per county was averaged prior to further analysis.

Historical monthly average temperature and precipitation data was obtained from the National Oceanic and Atmospheric Administration (NOAA),^2^ while historic monthly-average vegetative index was obtained from GRO-Intelligence.^3^ Vegetative index from GRO-intelligence was calculated from satellite data by taking into consideration multiple light spectra to enhance the presence of green vegetation by calculating normalized difference vegetation index (NDVI), which ultimately measures plant greenness. Historic meteorological data was linked to county level mycotoxin data by using the county and the year as common information. Originally, we obtained 1,386 data points of mycotoxin data for 99 counties, however, after linking toxin data with weather data this was reduced to 1,259 data points and 93 counties. The elimination of some data was due to the unavailability of enough historical average monthly weather data from NOAA for the following counties: Calhoun, De Witt, Grundy, Kendall, Livingston, and Mason.

### Features engineering and imputation for AFL data set modeling

All the monthly average precipitation and temperature data was averaged per county for the 14-years of historic data and for 2021. Average temperatures were calculated in degrees Celsius, and we used geographical centroids of each State (latitude and longitude) and the climate zones (Friedman, 2001). With these data, growth was calculated (Battilani et al., 2013) as described in equations 1 and 2. These equations have been applied to an European country with similar climate to Illinois (Van der Fels-Klerx et al., 2019).


A=5.98

B=1.70

C=1.43

T_max=48

T_min=5

(1) $$T_{\mathrm{eq}}=\left(T_{\mathrm{average}}-T\_\min\right)/\left(T\_\max-T\_\min\right)$$

(2) $$\mathrm{Growth}={\left[A\times \left(T_{\mathrm{eq}}{}^{B}\right)\times \left(1-T_{\mathrm{eq}}\right)\right]}^{C}$$

Where *T*_eq_ is calculated per month

Weighted growth (10% of original growth) was generated for the months where there is no corn in the field (January–April and November–December) and it is considered one of the assumption in our model. The aflatoxin production index was calculated (Battilani et al., 2013) using equations 3 and 4. These equations have been applied to an European country with similar climate to Illinois (Van der Fels-Klerx et al., 2019).


A=4.84

B=1.32

C=5.59

T_max=47

T_min=10

(3) $$T_{\mathrm{eq}}=\left(T_{\mathrm{average}}-T\_\min\right)/\left(T\_\max-T\_\min\right)$$

(4) $$\mathrm{AFLA}={\left[A\times \left(T_{\mathrm{eq}}{}^{B}\right)\times \left(1-T_{\mathrm{eq}}\right)\right]}^{C}$$

Where *T*_eq_ is calculated per month

We calculated the dispersal as an ON/OFF switch (Battilani et al., 2016) and assumed that dispersal was ON if there was less than 5 mm of accumulated rain per month. If there were more than 5 mm of accumulated rain per month we assumed that there was no dispersal (Battilani et al., 2013).

Finally, we engineered a featured named monthly aflatoxin risk index (ARI), during the months that there was corn in the field (Equation 5).


(5) ARI=growth×dispersal×afla

During the months where there was no corn in field (January–April and November–December) we calculated aflatoxin risk as described in equation 6 (Battilani et al., 2013; Van der Fels-Klerx et al., 2019). Weighted growth is an assumption in our model that takes only 10% of the fungal growth when there is no corn in the field.


(6) ARI=weighted_growth×dispersal

The inputs for the model were monthly ARI through the noted years for each county. We also added to our input features an average monthly vegetative index per county, which is a feature generated by satellite data and acquired from the GRO-intelligence company.

For any missing values in our monthly ARI or vegetative index features, we performed imputation using multivariate imputation by chained equations (mice; mean method was used; van Buuren and Groothuis-Oudshoorn, 2011) an R package (R Development Core Team, 2014). This imputation package allowed us to determine plausible data values that were created from the distribution of each missing data point (van Buuren and Groothuis-Oudshoorn, 2011). Due to the high number of missing data for ARI in January, we decided to remove this feature from the model instead of performing imputation. Finally, we linked the AFL data to the feature data set to create a set of 1,259 data points and 22 features or predictors. For FUM modeling, we could not use feature engineering functions such as the ARI calculated for AFL produced by *Aspergillus* because these equations were generated using *Aspergillus*.

### Weather data and imputation for FUM data set modeling

All the monthly average precipitation and temperature data were averaged per county for the 14-years of historic data, and for 2021. Average temperatures were expressed in degrees Celsius, and each county was assigned their geographical centroids (latitude and longitude), the climate zones (Friedman, 2001) and vegetative index. For any missing values in our monthly weather data or vegetative index features, we performed imputation using “mice” (mean method flag; van Buuren and Groothuis-Oudshoorn, 2011) an R package (R Development Core Team, 2014). Finally, we linked the FUM data to the feature data set to create a set of 1,259 data points and 33 features or predictors.

### Output variables and correlation analysis

We categorized the output values for AFL and FUM variables. For AFL a high category was considered for contamination levels greater that 20 ppb, medium for levels from 5 and 20 ppb, and low for levels 5 ppb or lower (Supplementary material 1).^4^ For FUM, a high contamination level was for values greater than 5 ppm, and the rest of the observations were low (Supplementary material 1). A correlation analysis was performed among all the predictors and output variables by using a confidence level of 0.95 for correlation and hclust method in R (R Development Core Team, 2014).

### Gradient boosting machine learning

Initial GBM analysis showed that ARI in November and December had zero influence in the model, therefore it was decided to remove engineering features and weather variables for the months of November and December which coincided with the end of harvest season in the State of Illinois. Removing these features and variables allowed the model to run through the corn growing year, that is, during pre-planting season, planting, plant growth/development, flowering time, and through harvest. The gbm software package in R was used, this software provided extensions to Freund and Schapire’s AdaBoost algorithm and Friedman’s gradient boosting machine (Friedman, 2001). For performing GBM, we first removed the Illinois county identifier from the data set, then partitioned the data for training and testing using a 70 to 30 ratio (Supplementary material 1). We performed GBM in AFL and FUM separately and during each individual mycotoxin analysis the other mycotoxin was removed from the data set. The input features (Predictors) used for AFL-GBM were the monthly ARI, and monthly veg. Index; the input features for FUM-GBM were the monthly PRCP, TAVG and veg. Index. For AFL we used the following flags on the training data: a threshold of 500 trees, interaction depth of one, shrinkage of 0.01, three cross validation folds and the distribution was selected as multinomial (Supplementary material 1). The gbm package was used to perform prediction analysis using the testing data set and the best fit generated from the training data. A confusion matrix was developed using the caret package in R that computed the overall statistics and the specific statistics by class. Finally, the gbm package was used to compute the effect values for each predictor in the model. For FUM, we used the following flags on the training data set: a threshold of 500 trees, interaction depth of one, shrinkage of 0.01, ten cross validation folds and the distribution was selected as multinomial (Supplementary material 1). The remaining analysis was performed as described for AFL.

### Bayesian network analysis

For BN analysis, we used the bn.learn package in R. The input features (predictors) used for AFL-BN were the monthly ARI, and monthly veg. Index; the input features for FUM-BN were the monthly PRCP, TAVG and veg. Index. First, we removed the climate zone and Illinois county input features from the data set, then discretized the full input features data set (not the latitude and longitude) by using the discretized R package with the cluster-method flag and 3 levels that were labeled as: L (Low), M (Medium) and H (High; Supplementary material 1). We partitioned the data for training and testing using a 70–30 ratio (Supplementary material 1). BN analysis for AFL and FUM was done separately and during each individual mycotoxin analysis the alternate mycotoxin was removed from the data set. The following analyses for AFL and FUM were done individually; first, we tested two structure building algorithms hill climbing (HC; Russell and Norvig, 1995) and TABU (Glover, 1986; Glover, 1989; Glover, 1990; Corazza et al., 2010) and performed scoring using the bayesian information criterion (bic-cg), then determined the classification error and visualized the conditional probability table for AFL and FUM (Supplementary material 1). The strength of the relationship was determined between BN nodes (input features) represented by each arc in the network to validate the structure, then tested the differences between HC and TABU BN topology. A Pearson correlation was performed if the number of connections was the same between BNs to determine if there were any differences in arc strength. Finally, we performed cross validation of the classifier using hill climbing and tabu with 10 runs each and the log-likelihood loss function (Supplementary material 1).

### Validation using 2021 mycotoxin data and GBM

The gbm software package in R was used to perform prediction analysis using the 2021 mycotoxin data set. Validation was done by using the best fit of GBM for AFL and FUM generated from the training data (14-years of historic data; Supplementary material 1). The weather and mycotoxin data for 2021 was prepared as previously described in the methods section. The mycotoxin data for year 2021 included 95 counties and a total of 371 observations.

R code: Available in Supplementary material 1.

## Results

### AFL and FUM contamination in Illinois

**Figure 1:**
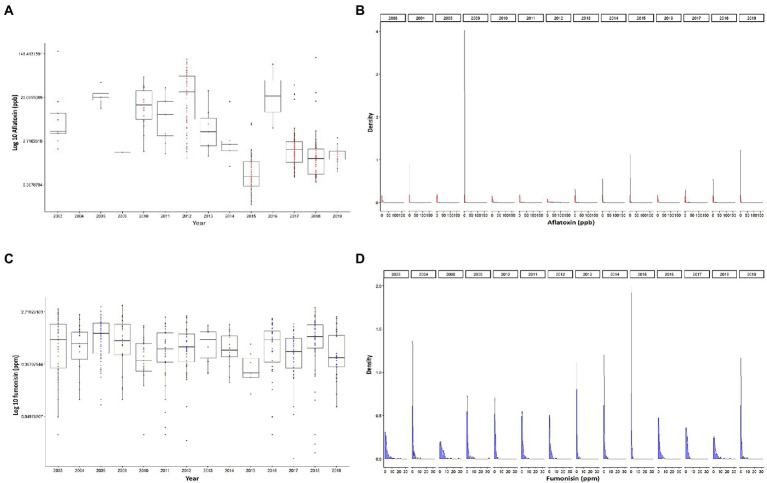
Distribution of mycotoxin contamination in Illinois. **(A)** Logarithmic base 10 scale of accumulation of AFL by year. **(B)** Density plots of logarithmic base 10 plus one of accumulation of AFL, faceted by year. **(C)** Logarithmic base 10 scale of accumulation of FUM by year. **(D)** Density plots of logarithmic base 10 plus one of accumulation of FUM, each panel represents a year. Red color depicts AFL, and blue color depicts FUM. Box-plot whiskers depict the maximum and minimum without outliers, and the box depicts median, first and third quantiles distribution.

In this study, we used 1,259 observations of corn mycotoxin contamination levels obtained from historic yearly survey summaries for the years (2003–2004 and 2008–2019) as determined and published by the Illinois Department of Agriculture. AFL data showed that only 4% of the samples had contamination levels >20 ppb, 3% medium levels (5–20 ppb) and 93% low level (<5 ppb). Thus, the data was zero inflated with high contamination levels >20 ppb being rare events (Figure 1). AFL contamination levels showed the highest recorded median levels in 2012 and the lowest in 2015 (Figure 1A). FUM contamination levels were not as variable as for AFL (Figure 1C), the data distribution showing 94% of data points were low (0–5 ppm), and 6% high (>5 ppm). We determined that the high percentage of low to zero levels of both AFL and FUM contamination (Figures 1B, D) indicated that the ability to detect high contamination levels would be based on rare events. This issue was considered during data preparation for machine learning analysis by keeping the training and testing data sets with a similar proportion of high/medium/low or high/low observations. In our analysis we decided not to use the same number of total cases for high/medium/low events because this would significantly reduce the number of total data points in the training set, thus decreasing the accuracy of the model.

The distribution of mycotoxin levels in relation to thermal climate zone in Illinois (Friedman, 2001) was examined. Climate zones vary in relation to the meteorological patterns, therefore there is a high correlation between climate zone and the geographical locations (latitude/longitude) of counties. In Illinois there are two climate zones, a mixed-humid climate zone, with more than 508 mm of annual precipitation and a monthly outdoor temperature below 7°C during the winter; and a cold climate zone, has 5,900 heating degree days per year (18.3°C basis; Baechler and Love, 2004). Our results showed an equivalent distribution of AFL levels between cold and mixed-humid zones while FUM contamination tended to be higher in mixed-humid environments (Supplementary Figure 1).

### Weather variables and feature engineering

**Figure 2:**
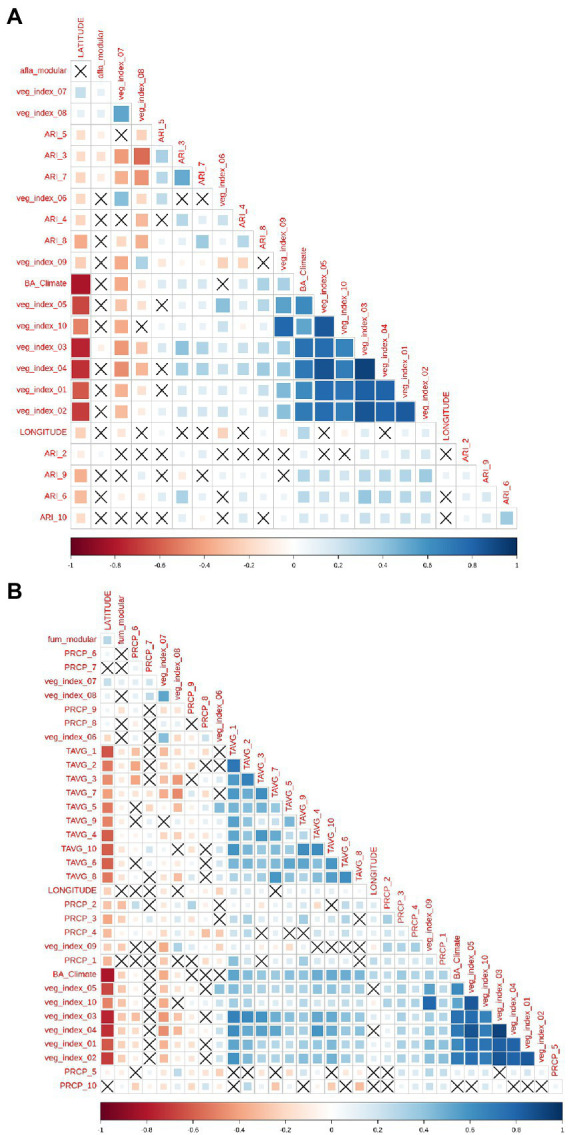
Pair-wise correlation analysis of all the model input variables. **(A)** Correlation analysis of the features used for aflatoxin modeling and **(B)**, for fumonisin modeling. Correlation level is depicted from positive correlation (blue) to negative correlation (red), black crosses represent non-significant *p*-values of correlation analysis between variables. *p*-value cut-off was 0.05 and confidence level 0.95.

For AFL, monthly aflatoxin risk indexes (ARI) were the main features engineered by employing mathematical functions that linked biological relationships between plant-fungal interactions (Battilani et al., 2013; Van der Fels-Klerx et al., 2019; Liu et al., 2021) with weather; for example, fungal growth (Equations 1 and 2), and patho-system interaction with the meteorological variables such as fungal dispersal (Battilani et al., 2013; Van der Fels-Klerx et al., 2019) (Equations 5 and 6). Feature engineering decreased the number of input variables that were incorporated into the model which also decreased the correlation levels among meteorological variables (Figure 2A). For FUM modeling, we could not use feature engineering functions such as the ARI calculated for AFL produced by *Aspergillus*. There were high correlation levels among the input variables used for modeling FUM (Figure 2B), nevertheless, the target variable, labeled fum_modular (Target variable = fumonisin), did not show high correlation levels with the input variables. In addition, the modeling algorithms used were designed to decrease overfitting caused by high correlation levels among variables. This low overfitting was accomplished because the basis of gradient boosting machine learning (GBM) is to sequentially generate model-ensembles that can learn from the errors of previous ensembles (Cooper, 1990; Friedman, 2001). A final input feature used for AFL and FUM models was vegetative index, this feature was obtained from satellite imaging which allowed us to include in the model the fluctuation in vegetation at the earth’s surface (Xue and Su, 2017).

### GBM analysis for AFL

**Figure 3:**
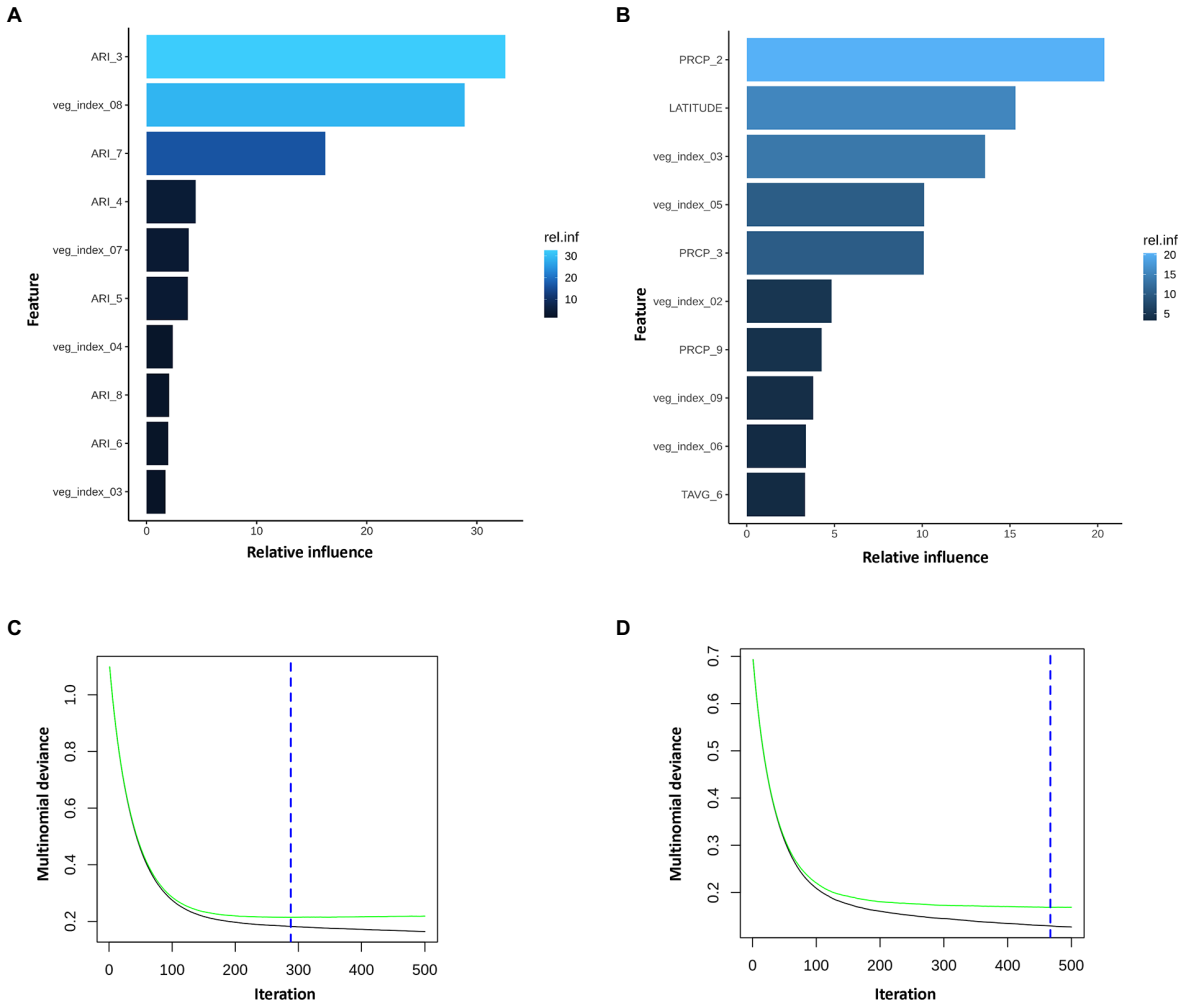
Summary of the GBM model using multinomial mycotoxin outcome. Top 10 influential input features and their relative influence over the model in prediction of **(A)**, AFL and **(B)**, FUM multinomial variables. Blue hue represents levels of relative influence of the input variables, light blue high and dark blue low influence level (AFL model had 19 of 22 features with non-zero influence; FUM model had 26 of 33 non-zero influence model). Multinomial deviance of **(C)**, AFL and **(D)**, FUM. Green line is testing set, black line training set and blue dotted vertical line is the number of iterations used (AFL, 288 iterations; FUM, 467 iterations). AFL model used interaction depth of 1, shrinkage of 0.01 and 3 c.v. folds and fumonisin depth of 1, shrinkage of 0.01 and 10 c.v. folds. FUM model used interaction depth of 1, shrinkage of 0.01 and 10 c.v. folds and fumonisin depth of 1, shrinkage of 0.01 and 10 c.v. folds.

We used GBM to model AFL contamination levels (factorial output variable) with our engineered features. Any features after harvest (October) were removed from the data frame. The model was able to predict the three contamination levels (high, medium, and low) (Table 1), and the optimal number of trees used was 288 which represents the number of trees at which the cross-validation error is minimized (Figure 3C). The McNemar *p*-value for the GBM models was 0.00129 (Table 2) which indicated that the proportion of type I and type II errors are not the same, possibly due to the differences in proportionality of high, medium, and low contamination levels in the prediction of the output variable. The overall accuracy of the GBM-AFL model was 94%, the class specific accuracy was 61% for high, 54% for medium and 60% for low contamination levels (Supplementary Table 2). The multi-class area under the curve was 0.5746 which evaluates the classifier in its ability to distinguish among classes.

**Table 1:** Confusion matrix of multinomial outcome class for AFL-GBM analysis to validate reference (testing data set) actual data for toxin levels and the predicted results using the model.

		Reference		
		High	Low	Medium
Prediction	High	3	1	0
	Low	10	349	10
	Medium	1	1	1

**Table 2:** Summary statistics of GBM and BN used for AFL and FUM.

	GBM		BN	
	AFL	FUM	AFL	FUM
Accuracy	0.94	0.94	0.94	0.94
95% Confidence Interval	(0.91, 0.96)	(0.91, 0.96)	(0.91, 0.96)	(090, 0.96)
McNemar’s Test *p*-Value	0.00129	0.0022	0.006324	2.668 × 10^−06^

The GBM model showed that from the 22 input features (predictors) only 19 had non-zero influence (Supplementary Table 1). Among the 19 features, the top 10 included ARI in March, February, April, May, June, July, and August, vegetative index in March, April, July, and August (Figure 3A; Supplementary Table 1). ARI in March was the feature with the highest influence in the model, which was unexpected because at that time of the year there is no corn growing in the field. To account for this, the fungal growth during the first 4 months of the year was weighted to lower values and aflatoxin production index was not included in the ARI calculation function (equations 5 and 6). To summarize, GBM model showed that March, a pre-planting month, had a strong influence over predictions for AFL contamination at harvest time. This observation provided a key to understand the environment-fungi-corn multi-way interactions that can be inferred from the predictions of the GBM model.

### GBM analysis for FUM

We used GBM to model FUM contamination levels (factorial output variable) by using average monthly temperature, precipitation, and vegetative index features. Features after the end of harvest (October) were removed from the data frame. The GBM model for FUM prediction using the testing data set, showed that the model was able to predict the two contamination levels of fumonisin (high, and low; Table 3). The optimal number of trees used was 467 which represents the number of trees at which the cross-validation error is minimized (Figure 3D). The overall accuracy of the FUM-GBM model was 94%, the class specific accuracy was for high was 58% (Supplementary Table 2) and the multi-class area under the curve was 0.577. These levels of accuracy are (considered) moderate and could be related to the detection rate per class that was imbalanced (McNemar’s test *p*-value was 0.0022, Table 2) due to the low frequency of high contamination values in the outcome variable.

**Table 3:** Confusion matrix of multinomial outcome class for FUM-GBM analysis to validate reference (testing data set) actual data for toxin levels and the predicted results using the model.

		Reference	
		High	Low
Prediction	High	4	4
	Low	20	349

The FUM model showed that from the 33 input features (predictors) only 26 had non-zero influence (Supplementary Table 1). Among the 26 features, the top 10 included precipitation in February, March, September, vegetative index in February, March, May, June, September, temperature in June, and latitude (Figure 3B; Supplementary Table 1). FUM-GBM models showed that precipitation in February was the most influential feature on the distribution of FUM contamination through the years in Illinois state, followed by latitude. Our model showed that counties below 41.5° latitude tend to be at be at higher risk to accumulate high levels FUM (Supplementary Figure 2). This latitude to mycotoxin relationship could be linked to geographical as well as meteorological differences in the State of Illinois, resulting in location-specific temperature, precipitation patterns.

### BN analysis for AFL

**Figure 4:**
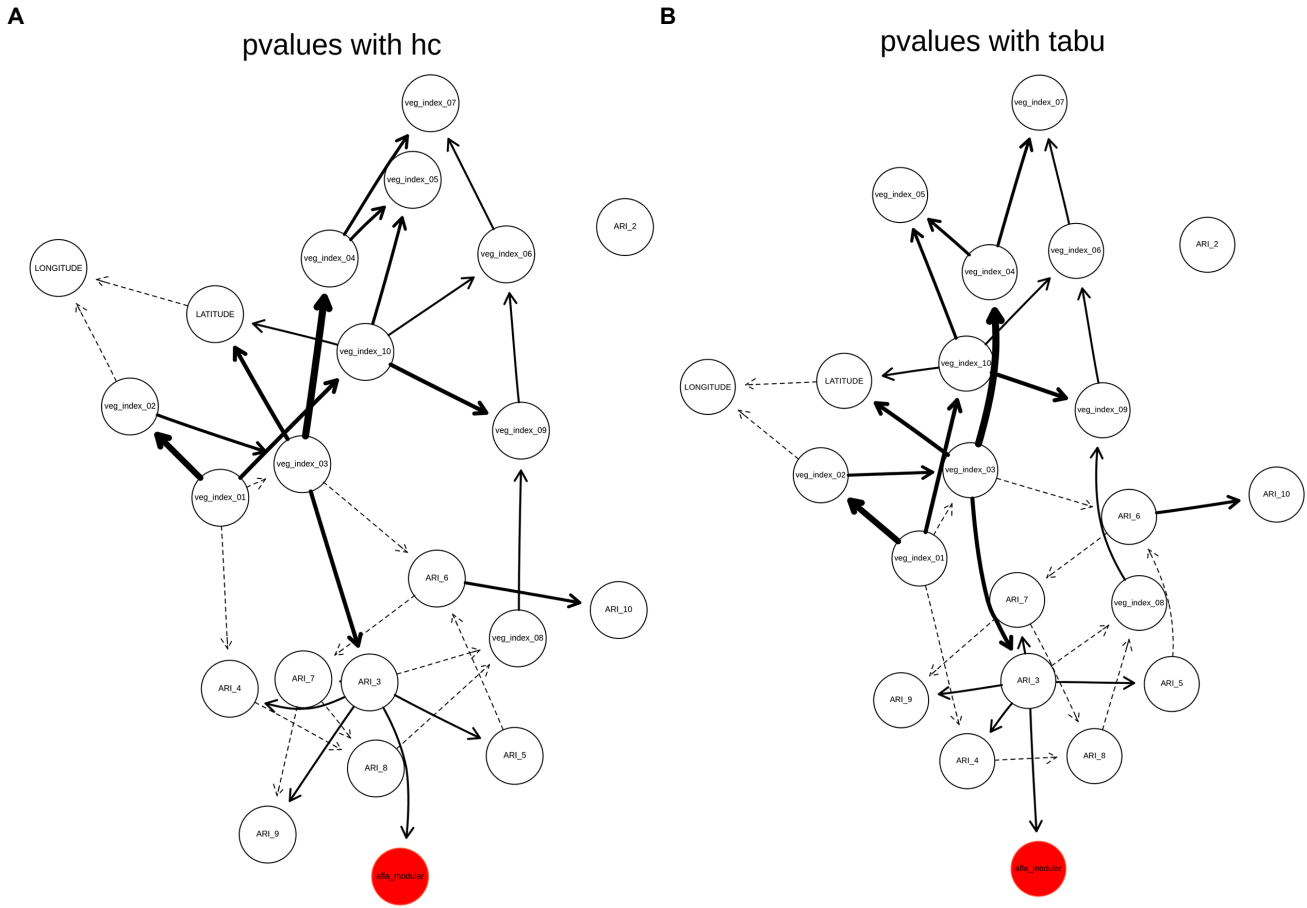
Weighted BN of AFL created by using **(A)**, hill-climbing (HC) and **(B)**, TABU algorithms. In the networks highlighted in red is the AFL node and in bold are significant interaction, thickness of the arrow represents strength of the interaction.

The weighted BN network results for AFL (Arc strength threshold >0.03) showed that the strength of connections between nodes are the same between HC and TABU algorithms (Figures 4A,B). In both HC and TABU derived networks, ARI in February is not part of the network (Figure 4), denoting this feature had no significant arc (Network connections) with any of the other features in the network. Both algorithm derived BNs showed the same parent node (ARI_3) for the target variable, afla_modular node (Figures 4A,B). The bayesian information criterion (bic) score of the two BNs showed no differences between using HC and TABU algorithms (−14003.7) and the log-likelihood loss values were almost the same when using TABU algorithm (15.2) compared to HC algorithm (15.1). The loss results allowed us to determine how close the predictions were when compared to the actual values. Because the difference in loss between TABU and HC-BN was small, the differences in the log-likelihood values insignificant, it is acceptable to use either network to predict AFL. The overall accuracy of both HC-BN and TABU-BN models was 94% (Table 2) and the class specific accuracy was 71% for high, 50% for medium and 63% for low levels (Supplementary Table 2).

### BN analysis for FUM

**Figure 5:**
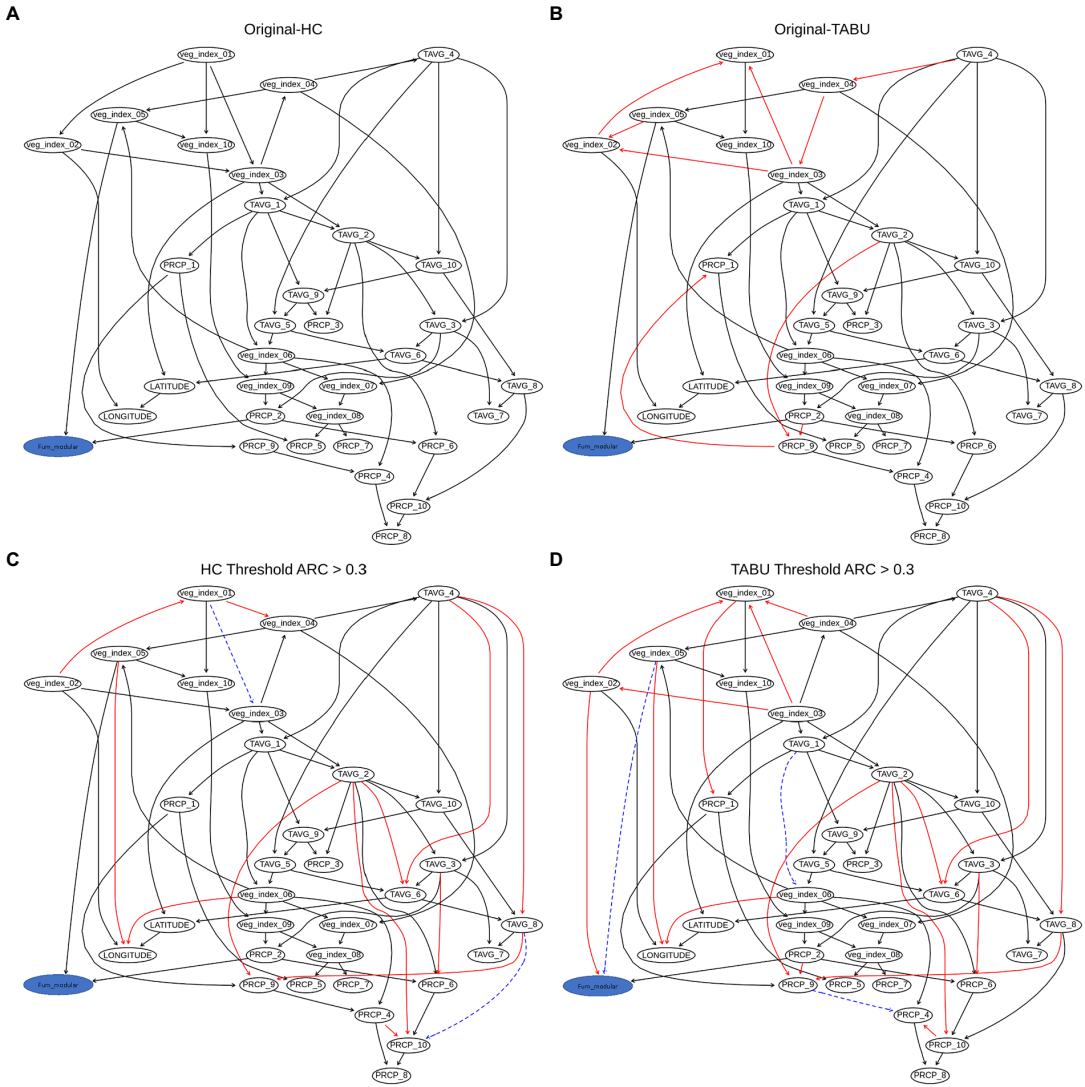
Weighted BN of FUM created by using **(A)**, hill-climbing (HC) and **(B)**, TABU algorithms. **(C)** Weighted network from HC evaluated using arc strength >0.3. **(D)** Weighted network from TABU evaluated using arc strength >0.3. The HC network was considered the baseline to which the other networks were compared, and the arrows are considered true positives, false positive arcs (which are missing or have different directions in the true network) are in red; false negative arcs are in blue and drawn using a dashed line.

The weighted network results for FUM-BNs (Arc strength threshold >0.03) showed differences in topology between HC and TABU derived networks (Figure 5). There were nine arcs present in HC-derived BN that were not present or had different directionality in TABU-derived BN (veg_index_02 to veg_index_01; veg_index_03 to veg_index_01; veg_index_03 to veg_index_02; veg_index_05 to veg_index_02; veg_index_04 to veg_index_03; TAVG_4 to veg_index_04; PRCP_2 to PRCP_9; PRCP_9 to PRCP_1 and TAVG_2 to TAVG_9) and six arc in TABU-derived BN compared to HC-derived BN (veg_index_03 to veg_index_04; veg_index_02 to veg_index_03; veg_index_01 to veg_index_02; veg_index_04 to TAVG_4; PRCP_1 to PCRP_9 and veg_index_01 to veg_index_03; Figure 5). HC, TABU derived BNs and weighted BNs, showed the same parent node for the fum_modular node, veg_index_05, and PRCP_2 (Figures 5). The bic score showed slightly lower value for TABU-BN (−24060.1) compared to HC (−24091.2.7) and the log-likelihood loss values were almost identical when using TABU algorithm (26.03) compared to HC algorithm (25.97). The overall accuracy of the TABU-BN model for FUM was 94% (Table 2). Due to the bic and log-likelihood values FUM TABU-BN would be better than FUM HC-BN at prediction of FUM levels.

### Model validation

To validate the model’s predictive capacity, we selected the GBM models generated for AFL and FUM because their overall accuracy and capacity to predict high levels of contamination was higher than FUM-BN models. Validation was done by using mycotoxin data from 2021, this data included 95 counties and a total of 371 observations. High levels of AFL contamination in 2021 were extremely rare, among the 371 observations only one had high levels of aflatoxin (0.3% incidence rate) and the rest were low levels of contamination. Number of observations of FUM contamination levels were 9 high (2%), 362 low (98%). The GBM models successfully predicted low contamination levels of AFL with an accuracy of 99% (Supplementary Table 3). The model was not able to correctly predict the single observation with high level of AFL contamination. For FUM-GBM, the model was able to successfully predict 1 out of 9 high contamination events, and 359 out of 362 low contamination events (Supplementary Table 3). The overall accuracy of FUM-GBM was 97% with a specificity of 99% and a balanced accuracy for high levels of 55%.

## Discussion

AFL and FUM contamination in USA corn has been a pervasive issue that leads to high economical loss in the agriculture sector (Vardon et al., 2003; Wu, 2006; Mitchell et al., 2016). In this USA-case study, we developed predictive models for AFL and FUM contamination by using historical mycotoxin data of corn contaminated with mycotoxins from the state of Illinois. This research represents the first single-State case study to develop predictive AFL and FUM models using machine learning algorithms in a major corn growing state in the USA. The predictive models developed herein showed greater than 90% overall accuracy. Furthermore, due to the nature of the GBM and BN models, it was possible for us to determine the input variables that significantly influence the models, this is a feature that other “black-box” type of ML models are unable to do, such as neural networks or support vector machine. Our input features analysis indicated that meteorological events prior to corn planting in the field strongly influence predictions of AFL and FUM contamination levels at harvest time. These type of early-year events detected by the models can directly assist farmers and stakeholders to make informed decisions on implementing interventions that prevent corn mycotoxin contamination in the field.

**Figure 6:**
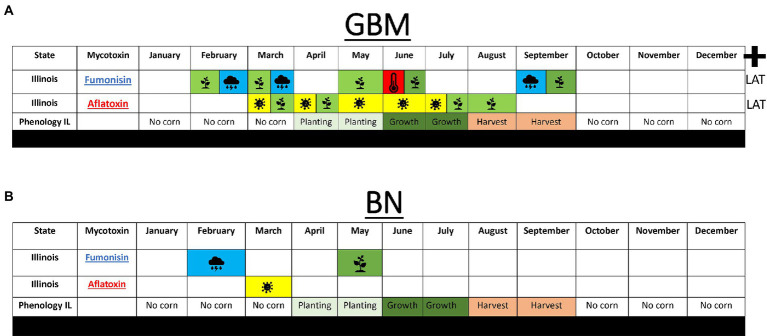
Summary of model analyses performed to predict fumonisin and AFL in Illinois. **(A)** Top 10 weather features for GBM using growth per month for AFL and **(B)**, BN using growth per months for AFL. Blue shade with rain cloud depicts average monthly precipitation, red shade with thermometer depicts average temperature, yellow shade with fungal head depicts average monthly fungal growth. Plus-sign indicates latitude was a significant feature in the model. Average phenology of cropping in Illinois is depicted as no-corn in the field (no color), planting time (light green), vegetative growth (dark green), harvest time (salmon) taken from Usual Planting and Harvesting Dates Agricultural Statistics Board from USDA National Agriculture Statistic Services (https://www.nass.usda.gov/).

Another unique feature in our models is that we used meteorological data including satellite acquired data to perform feature engineering, thus allowing us to combine mechanistic mathematical functions for AFL production (Battilani et al., 2013), *Aspergillus* growth and machine learning modeling approaches such as GBM (Friedman, 2001) and BN (Cooper, 1990). Similar type of weather associated input features and other machine learning approaches to predict mycotoxin contamination of corn have been used in Europe for AFL and FUM (Leggieri et al., 2021a; Wang et al., 2022), but to our knowledge, these approaches have not been used for prediction of mycotoxins in USA corn. Satellite generated data bases have been previously used in wheat studies in Europe and lead to higher model accuracy (Wang et al., 2022). Vegetative index derived from satellite data showed significant influence in the GBM models for both AFL and FUM (Figure 6; Supplementary Table 1) and in the BN model for FUM (Figures 5, 6). Additionally, our predictive models, which utilized 14 years of mycotoxin and meteorological data, represent one of the largest USA-based historical data sets used for modeling of AFL and FUM. A previous publication by Kerry et al. (2021) also used a large data set, however, it focused solely on AFL (Kerry et al., 2021).

The AFL and FUM-GBM models showed adequate overall accuracy and class specific accuracy. The measured class-specific accuracy levels were excellent when taking into consideration that, in the 14 year historical data, the data sets for AFL and FUM had 3 and 6% distribution of high contamination levels, respectively, thus the specificity of both models can be considered high (Supplementary Table 2). The observed overall and specific accuracy levels are at par when compared to other mycotoxin non-USA models that showed ranges from 90 to 99% general accuracy for wheat-models in Europe (Wang et al., 2022), 75% general accuracy for corn using AFLA-maize models in Italy (Battilani et al., 2013) and > 75% general accuracy using machine learning models for toxin prediction in corn in northern Italy (Leggieri et al., 2021a). A distinction comparing our models with these European published models, is that in our models we used high, medium, and low contamination levels for AFL and high/low for FUM, following the USA FDA^5^ regulations, which differ from the European standards. GBM was ideal to determine end of the year predictions that included all the input features used for training the model; while BN could update the probability distributions in the network without having to include in the test data set all the input features used in training the model. This indicated that BN allows predictions at any time point of the year while GBM only works at the end of the harvesting season (end of October).

In AFL and FUM-GBM models, latitude showed high influence on the contamination levels at the end of the year (Supplementary Table 1). Although, historically, AFL and FUM contamination levels exceeding 20 ppb and 5 ppm, respectively, have been relatively rare events (4 and 6%) in Illinois, those that have occurred have been in the southern part of the State (lower latitudes; Supplementary Figure 2). The historic data set revealed that the occurrence of corn FUM contamination levels exceeding 5 ppm were only found at latitudes below 41.5 (Supplementary Figure 2). Similarly, for AFL, 42° latitude is an apparent cut-off for 20 ppb (Supplementary Figure 2). Higher levels of FUM in relation to lower latitudes has been reported before from FUM levels measured in 17 locations in Illinois in 1990 (Shelby et al., 1994). Furthermore, forecasting models developed for Europe under different climate change scenarios have shown that, below 45° North latitude, aflatoxin risk production is higher than zero and in warmer temperature scenarios that geographical area expands further north (Battilani et al., 2016). We suggest that these latitude correlations with toxin levels are key historical factors to predict contamination toxin risk in the future because in Illinois topography, 40° latitude marks a geographical difference in the State that leads to differences in weather from cold to mixed-humid (Köppen, 2011; Beck et al., 2018). These latitude ranges are known to lead to meteorological differences which profoundly affected any prediction of mycotoxin contamination (Supplementary Figure 2) and could lead to optimal conditions for the fungi to produce more mycotoxins compared to higher latitudes if no major preventive measures are taken to mitigate crop risk.

BN analyses determined that weather and ARI for March are key determinants of AFL contamination levels at the end of the growth season. A study done with mycotoxin contamination in Serbia, created BN models for AFL and FUM using data from 2012 to 2016, found that early flowering time is a parent node of Aflatoxin contamination and later time periods of cob development is a parent node of fumonisin contamination (Liu et al., 2021). ARI during the months of early flowering time showed significant influence in our GBM models but not on our BN models. A key difference between our model and the Serbian model is that the time periods are different. We used February to October in monthly intervals, while the Serbian model used beginning of flowering time to harvest in eight sub-periods intervals (Liu et al., 2021). The overall accuracy for all our models was 94% (Table 2), and the GBM models showed higher class specific accuracy for FUM, meaning that GBM is recommended for AFL and FUM predictions. Model validation, using 2021 data and GBM models, showed higher overall accuracy levels compared to the predictions using the testing data sets (Supplementary Table 3), possibly due to the high specificity of the models to predict low levels of contamination. In 2021 there was low incidence of high levels of AFL (0.3%) and FUM contamination (2%). In the 14-year historic data set 4% (AFL) and 6% (FUM) of data points showed high levels of contamination. The differences in the percentages of high levels of contamination in the data sets makes prediction of this contamination class challenging. FUM-GBM was able to correctly predict one out of nine high contamination events, meaning that it performed better than AFL-GBM possibly due to the percentage of incidence differences in high contamination levels between AFL and FUM in 2021.

When comparing GBM and BN accuracies, we concluded that GBM performed better in determining AFL contamination at the end of the growing season. Due to the correlation levels among variables (Supplementary Figure 2), it is possible that GBM can deal better with these confounding factors. For both the AFL-GBM and FUM-GBM models, input features linked to March had high influence on the model, strongly suggesting that these two mycotoxins and their associated fungi might be influenced by weather parameters early in the year. Due to the influence that specific input features have on the models and the historical data distribution (Supplementary Figure 2), we concluded that prediction of high AFL contamination levels was linked to high ARI and high vegetative index in March. Correspondingly, prediction of high levels of FUM contamination were linked to high precipitation in February/March/September and high vegetative index in March and June (Supplementary Figure 2). It remains to be evaluated if differences between the diverse fungi *Aspergillus* (AFL producer) and *Fusarium* (FUM producer; Samapundo et al., 2005; Medina et al., 2017) in relation to growth, development and toxin production associated to environmental factors in the field are some of the underlying biological factors that leads to the differences in features influencing the GBM and BN models. There are agronomic practices that take place between corn growing seasons such as tilling and drilling that influence the levels of mycotoxin contamination (Borràs-Vallverdú et al., 2022). These factors associated with agronomic practices were not considered in our models, nevertheless, our models were able to determine that input features linked to between corn growing seasons have significant influence on the end of year mycotoxin contamination levels.

Previous models have shown that warmer weather early in the planting season (Early spring) leads to higher mycotoxin contamination levels in the USA (Yu et al., 2022). Our results indicate that weather between corn growing seasons significantly influence the contamination levels at the end of the year by leading to higher ARI. We theorize that the first months of the year can serve as “spring-growing-incubators” for the fungi. If environmental conditions (weather conditions or other agricultural practices) during the spring favor fungal growth (Borràs-Vallverdú et al., 2022), corn contamination levels at the end of the year will likely be high. Development of pest management strategies that take into consideration warmer and wetter spring seasons will be key to lower mycotoxin contamination in corn. In addition to climate, there are differences between *Aspergillus* and *Fusarium* in growth, development and toxin production associated to environmental factors in the field that lead to differences in implementing mechanistic-models to machine learning models aiming to predict AFL (Leggieri et al., 2021a; Wang et al., 2022) and/or FUM (Liu et al., 2021; Wang et al., 2022) contamination.

We also hypothesize that the meteorological, satellite-acquired features and environmental conditions linked to engineered features (derived using weather variables and mechanistic model functions) at the beginning of the year impacted fungal growth in soil residue or detritus of corn fields, resulting in significant contamination risk. Less granularity in weather parameters such as daily instead of monthly averages will help to increase model class specific accuracy and sensitivity, potentially leading to develop weekly predictive models. Further field experiments will be conducted to test the effects of weather over fungal growth in non-corn organic materials such as no-till fields. Advance prediction of weather ahead of the growing season for the corn growing areas will also assist us to determine any effects on corn production and the possibility of mycotoxin contamination. In conclusion, we have demonstrated from this study that it is possible to develop a predictive model for both AFL and FUM contamination based on monthly weather, satellite data and feature engineering with a high degree of accuracy – 94%. Model validation showed that the GBM models are highly specific and accurate to predict low contamination levels of AFL and FUM. Prediction accuracy for high levels of contamination is linked to overall incidence. We will, in future work continue to finetune these models using additional parameters such as daily weather and relative humidity data among other variables. In addition, we will be extending the modeling application to forecast contamination of corn with AFL/FUM in other corn growing states of the USA.

## Data availability statement

The datasets presented in this study can be found in online repositories. The names of the repository/repositories and accession number(s) can be found at: https://www2.illinois.gov/sites/agr/Plants/Mycotoxin/Pages/Survey.aspx.

## Author contributions

LC-D performed data analysis, imputation, feature engineering, modeling, contribution to biological relationships with the model, and manuscript preparation. JL and KR performed initial planning and support for collaborative work. MV, JL, KB and KR performed data collection, data preparation, contribution to biological relationships with the model and manuscript preparation. All authors contributed to the article and approved the submitted version.

## Funding

This work was supported by the US Department of Agriculture, Agricultural Research Service. USDA is an equal opportunity provider and employer.

## Conflict of interest

The authors declare that the research was conducted in the absence of any commercial or financial relationships that could be construed as a potential conflict of interest.

## Publisher’s note

All claims expressed in this article are solely those of the authors and do not necessarily represent those of their affiliated organizations, or those of the publisher, the editors and the reviewers. Any product that may be evaluated in this article, or claim that may be made by its manufacturer, is not guaranteed or endorsed by the publisher.

## Footnotes

^1^ https://www.wheatscab.psu.edu/

^2^ https://www.ncdc.noaa.gov/cdo-web/search

^3^ https://gro-intelligence.com/

^4^ https://www.fda.gov/food/natural-toxins-food/mycotoxins

^5^ https://www.fda.gov/food/natural-toxins-food/mycotoxins

## Abbreviations

AFL: Aflatoxins
FUM: Fumonisins
ARI: Aflatoxin risk index
GBM: Gradient boosting machine
BN: Bayesian network
FHB: *Fusarium* head blight
HC: Hill climbing algorithm
TABU: This is the name of an algorithm based on a Tongan word to indicate things that cannot be touched because they are sacred
BIC: Bayesian information criterion
Veg. Index: Vegetative index
PCRP: Precipitation
TAVG: Average temperature
