# Supplementary material for: Gradient boosting and bayesian network machine learning models predict aflatoxin and fumonisin contamination of maize in Illinois – First USA case study
Source: Front Microbiol. 2022 Nov 10;13:1039947. doi: 10.3389/fmicb.2022.1039947 (PMC9684211; doi:10.3389/fmicb.2022.1039947)
Supplement: Supplementary file 1 [file Data_Sheet_1.docx]

**Supplemental Material 1.** R code used for GBM and BN models.

#For GBM

#all_data is the data set of aflatoxin, fumonin, weather, ARI, vegetative index

all_data$afla_modular <- ifelse(all_data$Aflatoxin_Result_Avg_.ppb. > 20 ,

"High",ifelse(all_data$Aflatoxin_Result_Avg_.ppb.< 5, "Low", "Medium"))

all_data$fum_modular <- ifelse(all_data$Fumonisin_Result_Avg_.ppm > 5 ,

"High","Low")

sub_all$Aflatoxin_Result_Avg_.ppb.<-NULL

sub_all$Fumonisin_Result_Avg_.ppm<-NULL

#For aflatoxin

set.seed(1)

tmp1<-all_data %>% select(-c(fum_modular))

inTrain<-createDataPartition(y=as.factor(tmp1$afla_modular),

p=0.70, list = FALSE)

training <- tmp1[inTrain,]

testing<-tmp1[-inTrain,]

fit.gbm.1 <- gbm(training$afla_modular ~ .,

data = select(training,-afla_modular),

n.trees = 500,

distribution = "multinomial",

interaction.depth = 1,

shrinkage = 0.01,

cv.folds = 3,

n.cores=1)

#Find the best fit using cross validation

best.fit.gbm.1 <- gbm.perf(fit.gbm.1, method = "cv")

#Perform prediction using the testing data set

preds.gbm.1 <- predict.gbm(fit.gbm.1, testing, ntree = best.fit.gbm.1, type='response')

#Perform validation by doing prediction using the 2021 data set

preds2021.gbm.2 <- predict.gbm(object=fit.gbm.1 ,

newdata=Test_2021,

ntree = best.fit.gbm.1 ,

type='response')

#For fumonisin

set.seed(1)

tmp2<-all_data %>% select(-c(afla_modular))

inTrain2<-createDataPartition(y=as.factor(tmp2$fum_modular),

p=0.7, list = FALSE,times = 1)

training2 <- tmp2[inTrain2,]

testing2<-tmp2[-inTrain2,]

fit.gbm.2 <- gbm(training2$fum_modular ~ .,

data = training2,

n.trees = 500,

distribution = "multinomial",

interaction.depth = 1,

shrinkage = 0.01,

cv.folds = 10,

n.cores=1)

#Find the best fit using cross validation

best.fit.gbm.2 <- gbm.perf(fit.gbm.2, method = "cv")

#Perform prediction using the testing data set

preds.gbm.2 <- predict.gbm(fit.gbm.2, testing, ntree = best.fit.gbm.2, type='response')

#Perform validation by doing prediction using the 2021 data set

preds2021.gbm.2 <- predict.gbm(object=fit.gbm.2 ,

newdata=Test_2021,

ntree = best.fit.gbm.2,

type='response')

#####################################################################################

#####################################################################################

#For BN

#all_data is the data set of aflatoxin, fumonin, weather, ARI, vegetative index

set.seed(1)

all_data$afla_modular <- ifelse(all_data$Aflatoxin_Result_Avg_.ppb. > 20 ,

"High",ifelse(all_data$Aflatoxin_Result_Avg_.ppb.< 5, "Low", "Medium"))

all_data$fum_modular <- ifelse(all_data$Fumonisin_Result_Avg_.ppm > 5 ,

"High","Low")

sub_all$Aflatoxin_Result_Avg_.ppb.<-NULL

sub_all$Fumonisin_Result_Avg_.ppm<-NULL

tmp<-all_data %>% select(-c(LATITUDE, LONGITUDE,fum_modular,afla_modular,Illinois_County))

tmp2 <-tmp %>% mutate_if(is.numeric, funs(discretize(., method="cluster", breaks=3,labels =c("L","M","H"))))

tmp2$LATITUDE<-(sub_all$LATITUDE)

tmp2$LONGITUDE<-(sub_all$LONGITUDE)

tmp2$afla_modular<-sub_all$afla_modular

tmp2$fum_modular<-sub_all$fum_modular

#For aflatoxin

set.seed(1)

data<-select(tmp2,-fum_modular)

#data<-tmp2

inTrain<-createDataPartition(y=as.factor(data$afla_modular),

p=0.7, list = FALSE)

training <- data[inTrain,]

testing<-data[-inTrain,]

structure1 <- hc(training) #score-based structure learning algorithms

structure2 <- tabu(training) #score-based structure learning algorithms

bn.mod1 <- bn.fit(structure1, data = as.data.frame(training))

bn.mod2 <- bn.fit(structure2, data = as.data.frame(training))

pred1 <- predict(bn.mod1, node = "afla_modular", data=testing)

pred2 <- predict(bn.mod2, node = "afla_modular", data=testing)

pvalues1 = arc.strength(structure1, data = testing)

pvalues2 = arc.strength(structure2, data = testing)

xval.mod1 = bn.cv("hc", data = training, runs = 10)

xval.mod2 = bn.cv("tabu", data = training, runs = 10)

tructure1.1<-averaged.network(boot.strength(training, algorithm = "hc"), threshold = 0.3)

structure1.2<-averaged.network(boot.strength(training, algorithm = "tabu"), threshold = 0.3)

arcs(structure1.1) <- directed.arcs(structure1.1)

arcs(structure1.2) <- directed.arcs(structure1.2)

fitted1.1 = bn.fit(structure1.1, training)

fitted1.2 = bn.fit(structure1.2, training)

pred1.1 <- predict(fitted1.1 , node = "afla_modular", data=testing)

pred1.2 <- predict(fitted1.2 , node = "afla_modular", data=testing)

#For fumonisin

set.seed(1)

data<-select(tmp2,-afla_modular)

#data<-tmp2

inTrain<-createDataPartition(y=as.factor(data$fum_modular),

p=0.7, list = FALSE)

training <- data[inTrain,]

testing<-data[-inTrain,]

structure1 <- hc(training) #score-based structure learning algorithms

structure2 <- tabu(training) #score-based structure learning algorithms

bn.mod1 <- bn.fit(structure1, data = as.data.frame(training))

bn.mod2 <- bn.fit(structure2, data = as.data.frame(training))

pred1 <- predict(bn.mod1, node = "fum_modular", data=testing)

pred2 <- predict(bn.mod2, node = "fum_modular", data=testing)

pvalues1 = arc.strength(structure1, data = testing)

pvalues2 = arc.strength(structure2, data = testing)

xval.mod1 = bn.cv("hc", data = training, runs = 10)

xval.mod2 = bn.cv("tabu", data = training, runs = 10)

tructure1.1<-averaged.network(boot.strength(training, algorithm = "hc"), threshold = 0.3)

structure1.2<-averaged.network(boot.strength(training, algorithm = "tabu"), threshold = 0.3)

arcs(structure1.1) <- directed.arcs(structure1.1)

arcs(structure1.2) <- directed.arcs(structure1.2)

fitted1.1 = bn.fit(structure1.1, training)

fitted1.2 = bn.fit(structure1.2, training)

pred1.1 <- predict(fitted1.1 , node = "fum_modular", data=testing)

pred1.2 <- predict(fitted1.2 , node = "fum_modular", data=testing)
