# Supplementary figures and images for: Gradient boosting and bayesian network machine learning models predict aflatoxin and fumonisin contamination of maize in Illinois – First USA case study
Source: Front Microbiol. 2022 Nov 10;13:1039947. doi: 10.3389/fmicb.2022.1039947 (PMC9684211; doi:10.3389/fmicb.2022.1039947)

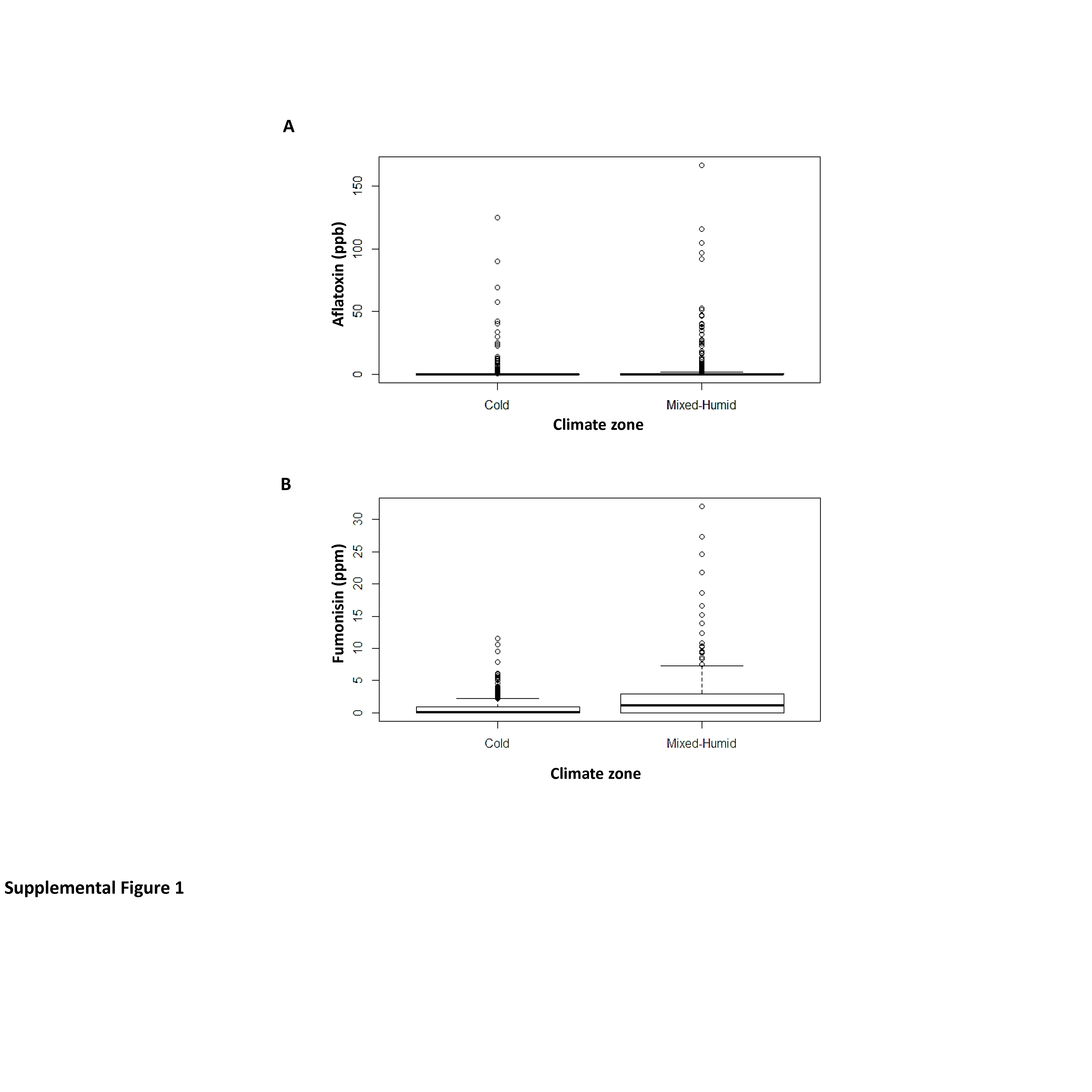

Supplement: Supplementary file 2 [file Image_1.tif]

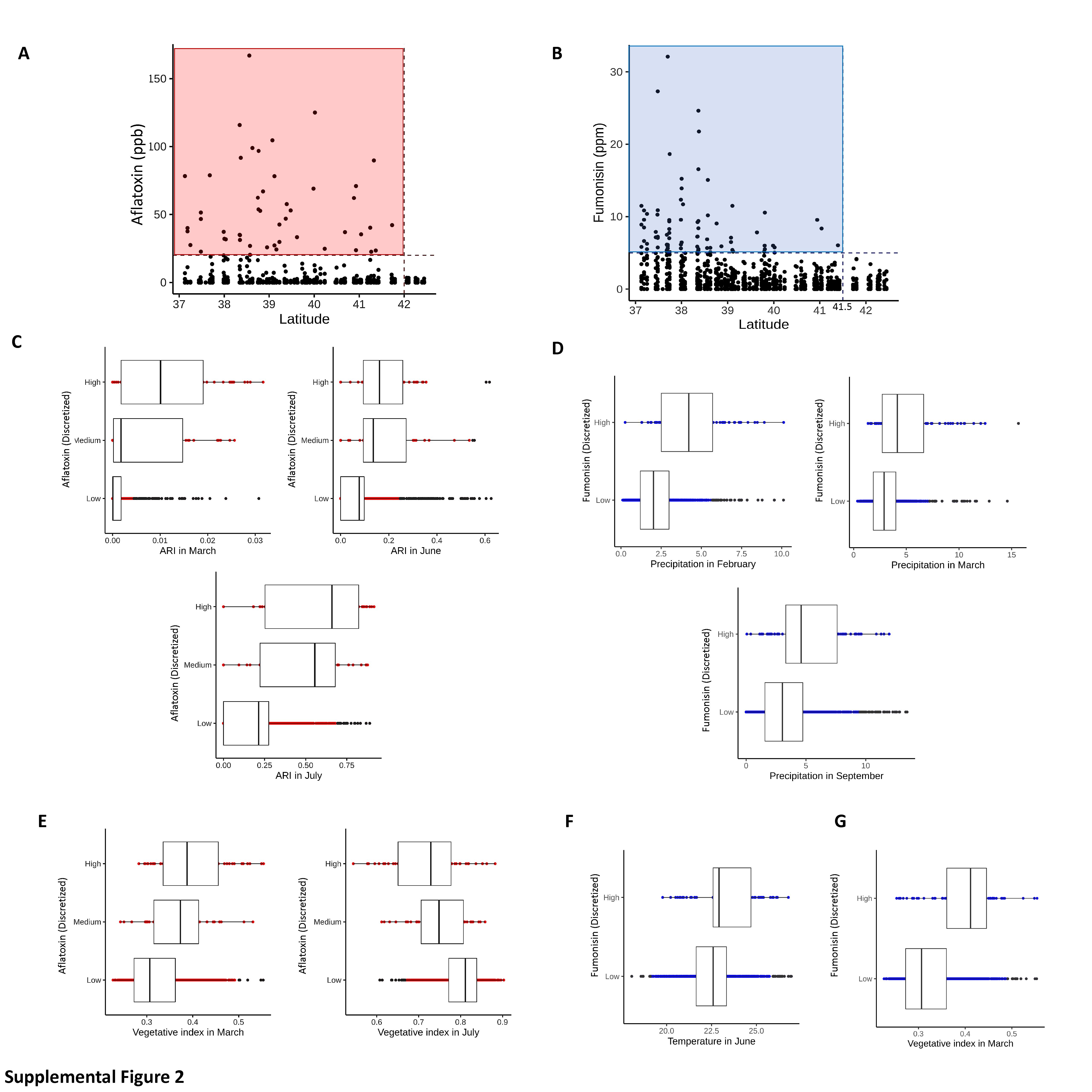

Supplement: Supplementary file 3 [file Image_2.tif]
